# Ultrasound-Assisted Ferritin Extraction from Northern Pike Liver: An Innovative Approach for Chlorogenic Acid Encapsulation with Enhanced Thermal Stability

**DOI:** 10.3390/molecules30092080

**Published:** 2025-05-07

**Authors:** Zhikun Xing, Yi Wang, Yabo Wei, Xin Guo, Xiaoyue Liang, Xiaorong Deng, Lianfu Zhang, Jian Zhang

**Affiliations:** 1 School of Food Science and Technology, Shihezi University, Shihezi 832003, China; xzk1109326642@163.com (Z.X.); 20222111021@stu.shzu.edu.cn (Y.W.); 18935813163@163.com (Y.W.); guoxin0317@shzu.edu.cn (X.G.); 18699146059@163.com (X.L.); dengxr22@shzu.edu.cn (X.D.); 2 Key Laboratory for Food Nutrition and Safety Control of Xinjiang Production and Construction Corps, School of Food Science and Technology, Shihezi University, Shihezi 832003, China; 3 Key Laboratory of Characteristics Agricultural Product Processing and Quality Control (Co-Construction by Ministry and Province), Ministry of Agriculture and Rural Affairs, School of Food Science and Technology, Shihezi University, Shihezi 832003, China

**Keywords:** northern pike (*Esox lucius*), ultrasound-assisted extraction, liver ferritin, structural characterization, homology modeling, embedding properties

## Abstract

Ferritin, an emerging protein resource, has garnered significant attention in scientific research due to its biocompatibility and unique cavity structure capable of encapsulating bioactive compounds. This study aimed to optimize ultrasound-assisted extraction (UAE) for enhancing ferritin yield from northern pike liver byproducts and evaluate its potential as a nanocarrier for chlorogenic acid (CA). Through response surface methodology (RSM), the optimal UAE parameters were established as 200 W ultrasonic power, 1:3 solid–liquid ratio, and 25 min extraction time. Under these conditions, the ferritin extraction yield reached 139.46 mg/kg, representing a 4.02-fold increase compared to conventional methods (34.65 mg/mL). Electrophoretic analysis confirmed the electrophoretic purity of the extracted liver ferritin. Comprehensive characterization using UV-vis spectroscopy, FTIR, and fluorescence spectroscopy revealed preserved structural integrity of UAE-extracted ferritin. Homology modeling provided molecular insights into the ferritin architecture. Successful encapsulation of CA was achieved with an encapsulation efficiency of 13.25%, as quantified by HPLC. Analysis by DLS and ζ potential as well as TG and DSC showed that not only the thermal stability of CA was enhanced after ferritin encapsulation, but also that the ferritin remained stable with a cage-like structure. This investigation establishes UAE as an effective strategy for valorizing fish processing byproducts through high-yield ferritin extraction while demonstrating the protein’s functional capacity as a nanocarrier for bioactive compound delivery. The findings highlight the dual advantage of sustainable resource utilization and advanced delivery system development through this biotechnological approach.

## 1. Introduction

Ferritin is a protein that stores iron and is extensively distributed in humans, plants, and animals [1]. Due to its nanoscale size and unique cage-like structure, it has increasingly attracted scholarly attention for the encapsulation, delivery, and controlled release of bioactive substances and drugs. Ferritin offers several advantages as a natural nanocarrier than other embedding systems. First, the encapsulated nanoparticles are small and homogeneous, typically measuring around 12 nm. Second, under physiological conditions, ferritin exhibits intrinsic targeting, low immunogenicity, excellent biocompatibility, and high intracellular stability in the bloodstream. Third, ferritin can dissociate into individual subunits under extreme pH conditions, specifically at pH values below 2 or above 11; upon neutralization, these subunits can reassemble into their original cage-like structure without altering properties. These features enable the ferritin’s inner cavity to effectively encapsulate and protect biologically active substances. Owing to the unique structure, superior physicochemical properties, and excellent biocompatibility of ferritin nanocages, their internal cavities are extensively utilized to encapsulate both inorganic and organic guest molecules. Notably, the encapsulation of bioactive molecules, such as curcumin, proanthocyanidins, and rutin, in ferritin nanocages has been shown to improve the bioavailability of these compounds [2] significantly.

Chlorogenic acid (CA) is a bioactive polyphenolic compound found mainly in many plants [3]. It is an effective neuroprotective agent and has antiviral [3], antifungal, antioxidant, and antitumor properties [4]. CA is also engaged in the regulation of glucose and lipid metabolism and may enhance insulin sensitivity [5]. Despite this, CA is vulnerable to the effects of exposure to light, heat, air, and enzymes used for digestion [6], and it is these environmental instabilities that limit its large-scale application, but its stability can be improved by exploiting the properties of ferritin’s inner-cavity encapsulation of bioactive substances [7].

The northern pike is a characteristic economic fish of Xinjiang, distributed in China exclusively in the Erqis River and Ulungu Lake. *Esox lucius* is flavorful, rich in protein, and of high nutritional value [8,9], typically transported as frozen fish after triple removal. The processing of northern pike typically results in 8–12% offal waste, which is high in protein content and, if not utilized, can lead to significant resource wastage. However, preliminary laboratory studies have identified the liver of *Esox lucius* as an ideal natural source of ferritin. Ultrasound is considered a low-cost and environmentally friendly auxiliary extraction method, and it enhances protein extraction efficiency by promoting mass transport and cell wall disruption [10]. Ultrasound-assisted extraction (UAE) significantly reduces solvent consumption and energy consumption through cavitation effect and parameter optimization, e.g., Wang et al. [11] achieved a 92% reduction in solvent consumption (liquid–solid ratio of 20:1 vs. 250:1) and a 96% reduction in time (1 h vs. 24 h) using ultrasound-assisted extraction of cordycepin, Hadi et al. [12] achieved a low liquid–solid ratio of 5:1 and a 50–90% reduction in solvent in extracting curcumin, and Poureini et al. [13] achieved a 50–90% reduction in solvent and extracted apigenin, with 50% reduction in solvent (25:1 vs. 50:1) and 91.7% reduction in time (30 min vs. 6 h). Farid Chemat et al. [10] reported that the energy consumption for ultrasound-assisted extraction of fats and oils from oleaginous seeds using different extraction methods required only 0.25 kW-h, which is 95.8% and 96.9% lower compared to solvent boiling point impregnation (6 kW-h) and soxhlet distillation (8 kW-h), respectively, and the extraction time was shortened from 8 h to a few minutes, which is a combination of high efficiency and decarbonization. These studies confirmed the performance of UAE in retaining the activity of target components (e.g., 86.45% purity of apigenin) while reducing solvent usage by 30–92% and time by 50–96%, which further validated its greening advantages. UAE enhances mass transfer through the cavitation effect, reduces energy consumption to 1.2 kWh/kg at room temperature, and has an energy-efficient conversion rate of 80–90%. Its optimized solid–liquid ratio to 1:3 (vs. conventional 1:4) reduces solvent requirement by about 25% while avoiding chemical additives. It has been demonstrated that UAE provides a sustainable solution for aquatic by-product resource utilization by combining high extraction efficiency (67.20%) with low carbon emission while maintaining the structural integrity of the proteins (e.g., retention of collagen triple-chain helices).

The efficiency of ultrasound-assisted extraction may be affected by a variety of factors such as temperature, solid–liquid ratio, time, and solvent type [14]. Response surface methodology (RSM) is an effective tool for finding the optimal conditions for a process when many factors and interactions may affect the desired response [15]. It has been extensively applied in developing various new protein resources, including porcine liver [15], duck liver [16], sesame seed bran [17], and sunflower seed meal [18]. Few studies have reported on the ultrasound-assisted extraction of fish liver ferritin. Previous studies established a process flow for extracting northern pike liver ferritin involving tissue cell fragmentation, heat treatment of the crude extract, neutral salt precipitation, and chromatographic separation. This method yielded a ferritin extraction of 34.65 mg/kg.

Although northern pike liver ferritin (NPLF) shows potential as a nanocarrier, its practical application still faces multiple challenges. In terms of scalability, natural NPLF relies on fish liver extraction, and there are industrialization bottlenecks of seasonal availability of raw materials and high purification costs (ultracentrifugation + chromatography). At the same time, recombinant expression systems need to solve the problems of assembly efficiency and stability [19]. In terms of bioavailability, NPLF lacks active targeting ligands, mainly relies on passive EPR effects, and is easily removed by the mononuclear phagocytosis system, and its pH-dependent disassembly properties may lead to premature drug release [20]. In terms of drug-carrying capacity, the size of the NPLF cavity (~8 nm) limits the loading of macromolecules, and its nucleic acid encapsulation efficiency is significantly lower than that of liposomes, which needs to be enlarged by gene editing or developed by surface-coupling strategies [21].

In the present study, we optimized the extraction process of northern pike liver ferritin using ultrasound-assisted techniques and successfully elucidated the spatial structure of NPLF through SDS-PAGE, Native-PAGE, UV spectroscopy, infrared spectroscopy, fluorescence spectroscopy, and homology modeling. This was ultimately applied for the encapsulation of chlorogenic acid. The study findings establish a theoretical foundation for the high-value exploitation of northern pike processing by-products, which are important for employing NPLF as a carrier of bioactive compounds in nutrient embedding and other applications.

## 2. Results and Discussion

### 2.1. Effect of Different Factors on the Amount of Ferritin Extracted from the Liver of Northern Pike

**Figure 1:**
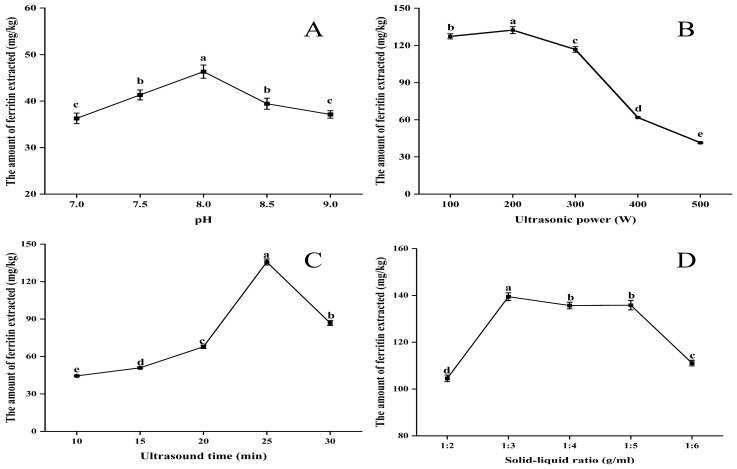
A one-factor experiment on liver ferritin in the northern pike (**A**) pH (**B**) ultrasonic power (**C**) ultrasonic time (**D**) solid–liquid ratio. Different lowercase letters indicate significant differences (*p* < 0.05) among treatments. Significant correlation analyses are detailed in the Supplementary Materials. Note: Images of the buffer types are shown in the accompanying Figure S1.

Figure 1A illustrates that the extraction of ferritin exhibited an initial increase followed by a decrease as pH increased, with a peak value of 46.35 mg/kg observed at pH 8.0. The pH of the extract at this time significantly deviates from the isoelectric point of NPLF [22], thereby enhancing protein solubilization. Under these conditions, the binding affinity of NPLF to phosphate is enhanced, which further facilitates proteolysis.

As shown in Figure 1B, initially, as the ultrasound power increased from 100 W to 200 W, the interaction between water and protein molecules was enhanced, leading to an increase in ferritin extraction [23,24]. This is due to the cavitation and mechanical effects induced by ultrasound, which promotes the hydrolysis of proteins. However, when the ultrasound power is continuously increased from 200 W to 500 W, the excessive power may lead to aggregation of solubilized protein molecules, thus reducing the rate of increase in ferritin extraction.

It is undeniable that the duration of ultrasonic extraction significantly influences the protein extraction process. As illustrated in Figure 1C, extending the extraction time from 10 to 30 min resulted in a peak protein extraction of 135.68 mg/kg at 25 min. However, the amount of ferritin extracted began to decline as the time increased to 30 min. This phenomenon is understandable, as an appropriate extraction time facilitates the dissolution of proteins from the raw material. Excessive ultrasonic extraction time can lead to protein aggregation and denaturation, resulting in reduced protein extraction [25].

Ferritin extraction rapidly increased to 139.46 mg/kg as the feed-to-liquid ratio increased from 1:2 to 1:3 (Figure 1D). As the ratio continued to increase to 1:4, ferritin extraction began to decrease gradually, and by 1:6, it had decreased significantly. This result can be explained by the following mechanism. When the same buffer was used to extract ferritin, the amount of ferritin extracted increased gradually with the increase in the material–liquid ratio, but the increase in ferritin extraction slowed down when the material–liquid ratio exceeded 1:3, indicating that a certain amount of buffer was favorable to the dissolution of ferritin in the livers of the northern pike under the effect of ultrasonic cavitation. The appropriate solid–liquid ratio made the homogeneous liver raw materials fully accept the cavitation force and strongly collide with each other to improve the dissolution and extraction rate of ferritin, while higher or lower buffer amounts will prevent the proteins in the liver from being completely dissolved, thus affecting the cavitation effect and reducing the ferritin extraction rate. Therefore, 1:3 (*w*/*v*) was chosen as the optimal extraction material–liquid ratio [26]. Also, this may be due to the ultrasonic cavitation effect, which optimized the optimal solid–liquid ratio from 1:4 to 1:3 in the conventional thermal extraction method by enhancing the mass transfer efficiency, and the solvent dosage was reduced by 25% compared to the conventional method, which reflects the high efficiency and environmentally friendly characteristics of the ultrasound-assisted extraction method [25].

### 2.2. Optimization of Ferritin Extraction Process by RSM Method

**Figure 2:**
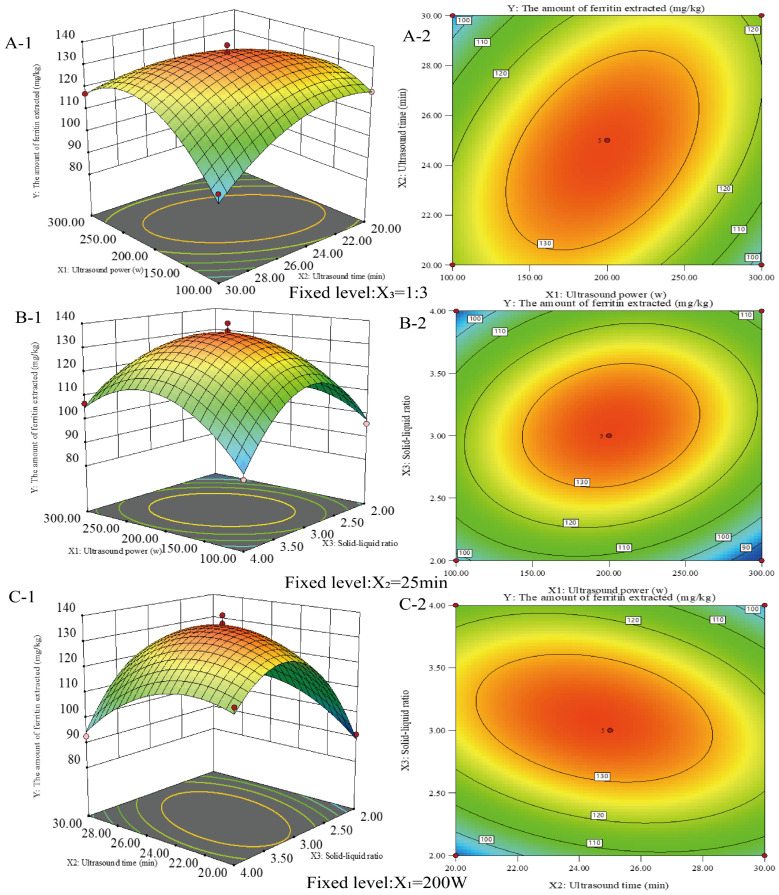
Response surface plots (**A**-**1**, **B**-**1**, and **C**-**1**) and contour plots (**A**-**2**, **B**-**2**, and **C**-**2**) showing the interaction effect of ultrasonic power (X_1_), ultrasonic time (X_2_) and solid–liquid ratio (X_3_) on the amount of ferritin extracted from the livers of the northern pike with ultrasonic-assisted extraction.

The response surface technique was used to optimize the circumstances in which ferritin was extracted from the liver of the northern pike. As shown in Figure 2, the extraction process was further optimized using a Box–Behnken design, considering ultrasound power (X_1_), ultrasound time (X_2_), and material-to-liquid ratio (X_3_) as influencing factors. The protein extraction amounts under the three-factor, three-level factorial design are presented in Table 1. Protein extraction ranged from 84.22 mg/kg to 139.46 mg/kg. The maximum ferritin extraction of 139.46 mg/kg was achieved at an ultrasonic power of 200 W, extraction time of 25 min, and a material-to-liquid ratio of 1:3. Regression analyses were conducted to assess the effects and potential interactions of the factors, as well as to evaluate the statistical significance of the model. The regression equation for protein extraction rate (Y) is

Y = +135.65 − 0.16X_1_ − 1.38X_2_ + 3.92X_3_ + 11.65X_1_X_2_ + 7.56X_1_X_3_ − 8.64X_2_X_3_ − 17.74 X_1_^2^ − 10.85 X_2_^2^ − 24.34 X_3_^2^ (1)

Table 2 indicates that the model response fit is significant, suggesting that the model effectively describes the relationship between the response and the factors. Three linear (X_1_, X_2_, X_3_), three quadratic ($x_{1}^{2}$, $x_{2}^{2}$, $x_{3}^{2}$), and three interaction terms (X_1_X_2_, X_1_X_3_, X_2_X_3_) were significant (*p* < 0.05). The coefficient of determination (R^2^) was 0.9845, indicating that the quadratic regression model explained 98.45% of the total variation. The results indicated that the optimal extraction process for ferritin from the liver of Northern Pike yielded up to 139.46 mg/kg at 200 W ultrasound power, a 1:3 feed-to-liquid ratio, and a 25 min ultrasound time.

### 2.3. Homology Modeling of the NPLF

**Figure 3:**
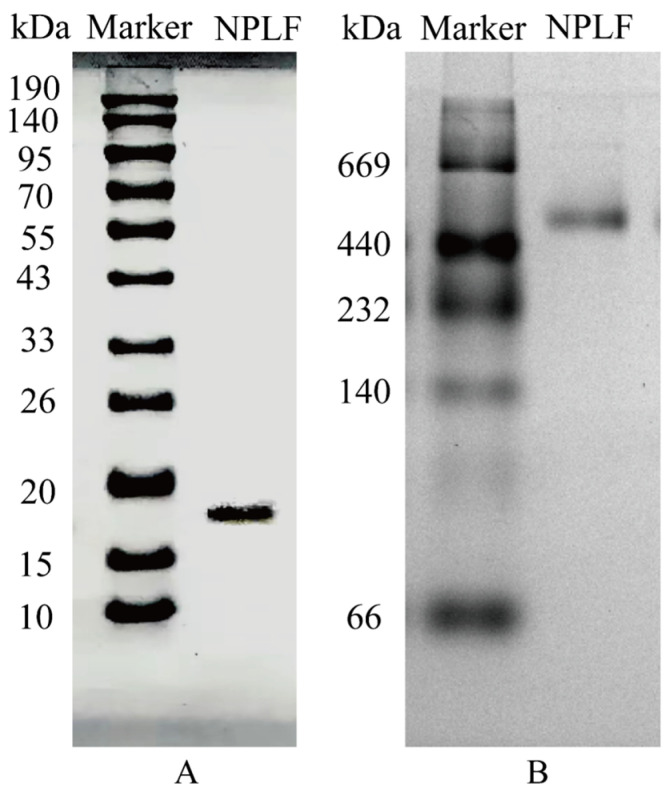
SDS–PAGE and Native PAGE analyses of northern pike liver ferritin (NPLF). (**A**) SDS-PAGE. (**B**) Native-PAGE. Lanes M represents protein markers and lanes NPLF represents the purified NPLF sample.

**Figure 4:**
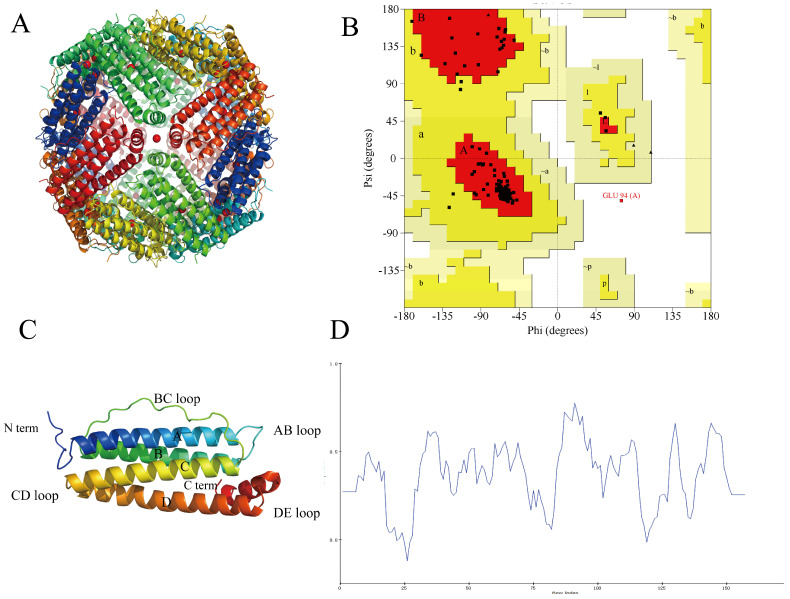
Homologous modeling of NPLF. (**A**) Schematic representation of liver ferritin in northern pike. (**B**) Ramachandran plot of NPLF subunit. ψ and φ represent Psi and Phi, respectively. In the picture, A, B, and L are the most favored regions; a, b, l, p are additional allowed regions; ~a, ~b, ~l, ~p are generously allowed regions and others are disallowed regions. (**C**) The tertiary structure of NPLF subunit by homologous modeling. A, B, C, D, and E represent five helixes, respectively. (**D**) The verify-3D plot of NPLF. At least 80% of amino acid residues should have a score greater than or equal to 0.2. (Figure 4D of the associated high-resolution image in Supplementary Figure S2).

The subunit sequence of NPLF was determined using gel digestion (gel electrophoresis results are shown in Figure 3) and subsequently characterized using LC-MS/MS. According to a mass spectrometry study, 84.65% of the amino acid sequence of NPLF matched northern pike ferritin A0A3P9AQF8 [27]. Table 3 demonstrates that NPLF matched 10 amino acid sequences, thereby confirming the identification of the protein as northern pike ferritin and validating the success of the purification process. The results of homology modeling are presented in Figure 4.

The protein cage structure and modeling of the subunits of NPLF are shown in Figure 4A,C. The structure of the NPLF subunit consists of four antiparallel α-helices (A, B, C, D), one short α-helix (E), and four loops (AB, BC, CD, DE), similar to that of the previously reported phytoferritin. The Ramachandran plot (Figure 4B) visualizes the dihedral angles ψ and φ of the amino acid residues of the main chain, in which A, B, and L are the most favorable regions; a, b, l, and p are the reasonable regions; ~a, ~b, ~l, and ~p are the permissive regions; and the others are the impermissive regions. 94.6% of the amino acid residues of the NPLF are located in the most favorable regions, and 5% are located in the permissive regions, and only 0.4% of amino acid residues are located in the impermissible region. Therefore, the principle of the evaluation that the reasonable zone plus the permissible zone is greater than 90% indicates that the model is more reasonable, and the structure of the protein obtained by the construction is more reliable and can be used as a template for subsequent research. Verify-3D is a model evaluation procedure based on the ‘threading’ method, which uses the scoring function to detect the relationship between the constructed model and its amino acid sequence, and the validation score of 80% residues is greater than 0.2, which is reasonable. As shown in Figure 4D, the average score of 86.63% amino acid residues in the protein structure in Verify-3D is greater than 0.2, which indicates that the protein model has good credibility.

Numerous studies have shown that the subunit N-terminal region of phytoferritin has a unique structural domain extension peptide (EP), which participates in the oxidative deposition of Fe^2^⁺ through conserved histidine residues and regulates the dynamic release of iron nuclei through conformational changes [28]. However, the endogenous serine protease activity of EP leads to the susceptibility of plant ferritin to autohydrolysis during long-term storage, significantly reducing its conformational stability. Unlike plant ferritin, animal-derived ferritin avoids the risk of autohydrolysis due to the lack of the EP structural domain and may have higher stability and carrier capacity [29].

In the present study, it was shown by SDS-PAGE and Native-PAGE analysis that NPLF consists of subunits with a molecular weight of about 19 kDa, and the natural oligomer has a molecular weight of 480 kDa (Figure 3A,B), which is in agreement with that reported for oyster ferritin [30]. Further comparisons revealed that, compared with plant ferritin, the NPLF subunit lacks the EP structural domain of plant ferritin (schematically shown in Figure 4C), which circumvents subunit degradation triggered by autohydrolysis; and that, compared with homologous animal ferritin (e.g., oyster ferritin), differences in the subunit sequences may lead to differences in acid resistance and iron-binding capacity, despite the similar molecular weights of the oligomers (~480 kDa) [27].

### 2.4. Preparation of Apo-CA Nanoparticles

**Figure 5:**
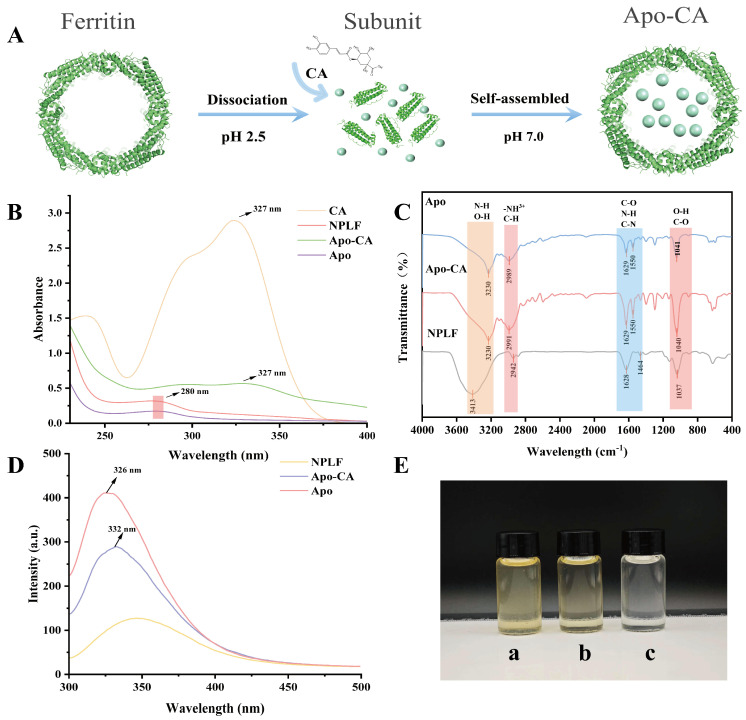
Characterization of northern pike liver ferritin NPLF and Apo ferritin (Apo) and encapsulated chlorogenic acid Apo-CA nanoparticles. (**A**) Schematic representation of NPLF encapsulated chlorogenic acid. (**B**) UV-vis spectra of NPLF, Apo, and Apo-CA nanoparticles. (**C**) FTIR spectra of NPLF, Apo, Apo-CA nanoparticles. (**D**) Fluorescence spectra of NPLF, Apo, Apo-CA. (**E**) Color change in NPLF (a) before purification, NPLF (b) before purification, Apoferritin (c).

As shown in Table 4 and Table 5, ferritin achieved a 94.58% iron removal rate, making it suitable for subsequent experiments. The successful preparation of Apo was confirmed by the fact that the ferritin solution was reddish-brown before iron removal and became colorless thereafter (Figure 5E). Figure 5E shows the NPLF solution before purification, b shows the NPLF solution after purification, and c shows the Apo solution. It can be seen that the NPLF solution has a yellow color due to the large amount of Fe^3+^ in it, whereas the Apo solution is colorless and transparent because it contains almost no iron ions. The solution color changed from tan to colorless (Figure 5E), indicating the successful removal of iron nuclei. Combined with ICP-MS analysis, TEM structural imaging, and high-performance liquid chromatography analysis, it was confirmed that Apo maintained its intact structure (~12 nm) and functional stability after iron removal (successfully encapsulated chlorogenic acid by utilizing reversible self-assembly property). Utilizing the pH-triggered self-assembly characteristics of ferritin nanocages (as shown in Figure 5A), the bioactive compound CA was successfully encapsulated in apoferritin (Apo-CA).

### 2.5. UV Spectral Analysis of NPLF, Apo, and Apo-CA

UV-visible absorption spectra (Figure 5B) elucidated the structural features and interaction mechanisms of ferritin, apolipoprotein (NPLF, Apo), and its chlorogenic acid complexed form (Apo-CA). Natural ferritin (NPLF) has an atypical UV-vis absorption spectrum (230–400 nm) [31]. Despite the presence of aromatic amino acids (tyrosine, tryptophan, and phenylalanine) in its proteashell, characteristic absorption peaks associated with π → π* electron leaps are not readily recognizable at 280 nm. This anomaly is attributed to the predominant absorption of the iron nucleus (Fe^3^⁺-phosphate complex) in the UV region, which masks the intrinsic absorbance of the protein shell and leads to a monotonically decreasing absorption profile. Iron removal restored the typical protein absorption properties of desferrin (Apo), especially the 280 nm peak, confirming the masking effect of the iron core and demonstrating that iron removal eliminated spectral interferences, allowing accurate discrimination of the absorption properties of aromatic amino acids. After chlorogenic acid encapsulation (Apo-CA), the UV spectra show a double-peak feature: the 280 nm peak is red-shifted, indicating a change in the polarity of the microenvironment due to the binding of aromatic residues (e.g., tryptophan and tyrosine); a new peak at 327 nm corresponds to the π → π* transformation of the phenolic hydroxyl group in chlorogenic acid, which confirms the successful doping of ferritin nanocapsules [32].

### 2.6. NPLF, Apo, and Apo-CA Infrared Spectral Analyses

FTIR spectra (Figure 5C) showed that the characteristic peaks of Apo included 3230 cm^−1^ (amide A band, N-H and hydrogen bonding vibration), 2989 cm^−1^ (carboxylic acid C-H vibration), 1629 cm^−1^ (amide I band, C=O stretching vibration) and 1550 cm^−1^ (amide II band, N-H bending and C-N vibration) and 1041 cm^−1^ (O-H deformation and C-O stretching vibration), suggesting that the ferritin conformation is intact [33]. The amide I/II bands in the Apo-CA complex were only slightly shifted, suggesting that the secondary structure of ferritin is less interfered with by chlorogenic acid (CA) and the appearance of the characteristic peaks of CA (1629 cm^−1^ and 1550 cm^−1^) indicated that CA was successfully encapsulated [34]. A shift in the characteristic peaks associated with NPLF was observed compared to Apo, which may be because the iron nucleus has not been removed yet. In addition, the change in the intensity of the peak in the hydroxyl/amino region (3242/3290 cm^−1^) suggests that CA is bound to the ferritin surface groups through hydrogen bonding, which is consistent with the enhanced transparency of the tryptophan microenvironmental poi in the fluorescence spectra. The above peak attribution elucidates the mechanism of CA interaction with ferritin and supports the stability of the encapsulated complex.

### 2.7. NPLF, Apo, and Apo-CA Fluorescence Spectral Analysis

It was shown that tryptophan in ferritin is mainly located in the 4-fold channel and is formed via E-helix. Based on this property, different samples were analyzed using fluorescence spectroscopy. As shown in Figure 5D, Apo showed high fluorescence intensity at 326 nm, which was significantly higher than that of the iron-containing nucleus of NPLF, indicating that the iron nucleus exerts a fluorescence bursting effect on tryptophan (Trp) through the mechanism of electron transfer or resonance energy transfer, which is in agreement with that of the reported for the shark liver ferritin (SZLF) and the horse spleen ferritin (HSF) [35]. After encapsulation of chlorogenic acid (Apo-CA), the emission peak was red-shifted to 332 nm, and the fluorescence intensity was further reduced, suggesting that chlorogenic acid enters the 4-fold channel through hydrophobic interactions or hydrogen bonding and binds to Trp near the E-helix, leading to enhanced polarity of its microenvironment. The red shift is consistent with the range of conformational changes induced by ligand binding in the literature, confirming the specific binding of chlorogenic acid to ferritin.

### 2.8. Transmission Electron Microscopy Analysis

**Figure 6:**
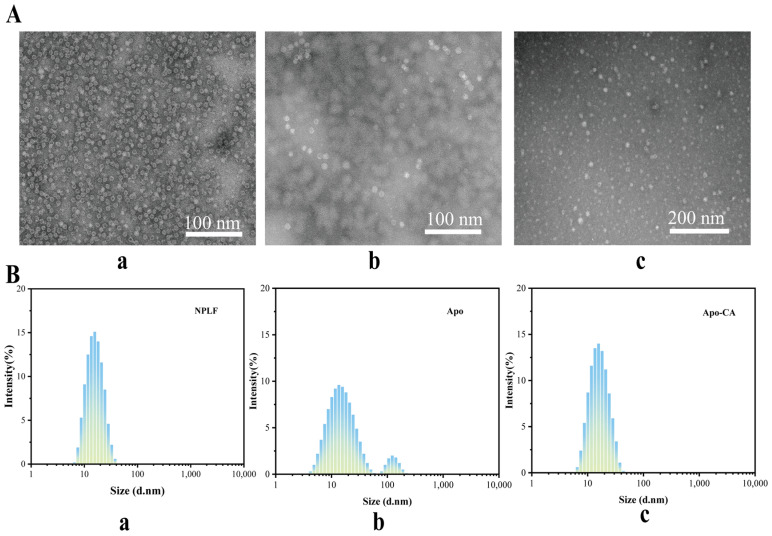
Transmission electron microscopy and particle size analysis of liver ferritin in northern pike. (**A**) Transmission electron microscopy of NPLF (**a**), Apo (**b**), Apo-CA (**c**). (**B**) Particle size distribution of NPLF (**a**), Apo (**b**), and Apo-CA (**c**).

The investigation of the sample’s microstructure is made possible by the use of transmission electron microscopy (TEM). As seen in Figure 6A, phosphotungstic acid enters the ferritin’s inner cavity with an average size of about 12 nm, creating a black uranium-filled sphere-like structure inside. This is consistent with the observations that have been previously published [36]. Both NPLF and Apo were negatively stained with phosphotungstic acid and then examined using TEM, with results presented in Figure 6A. Figure 6A shows that NPLF is a globular molecule consisting of a low-electron-density protein shell and a high-electron-density iron core, b is a TEM micrograph of Apo-CA, and c is a TEM micrograph of Apo-CA nanoparticles. The iron nuclei in NPLF were removed after confined reduction dialysis, resulting in the disappearance of the black high-electron-density iron nuclei in Apo. Only the light-colored, low-electron-density protein shells remained, with no significant changes in the shape and size of NPLF and Apo, suggesting that the iron removal process did not affect the ferritin structure. Figure 6A displays the TEM micrograph of Apo-CA nanoparticles, where the reappearance of a black core in the Apo cavity indicates that CA molecules filled the cavity, confirming the successful encapsulation of CA within the ferritin’s inner cavity.

### 2.9. Particle Size Analysis

Figure 6B presents the particle size distributions of NPLF, Apo, and Apo-CA. The particle size of natural NPLF (with iron core) is about 12 nm (Figure 6(Ba)). Two peaks appeared in the particle size of Apo (without iron nucleus) after iron removal (Figure 6(Bb)), with the primary peak still having a particle size of about 12 nm and the secondary peak (~10%), corresponding to a small number of aggregated particles (with a particle size of more than 100 nm). The formation of these aggregated particles of Apo may be due to the iron nucleus removal effect: the endogenous iron nuclei may inhibit the dissociation of subunits through electrostatic shielding, and the inter-subunit repulsion is enhanced after the removal of iron, which leads to some aggregated ones being formed [37]. The particle size distribution of Apo-CA nanoparticles, after encapsulating chlorogenic acid, is also more uniform, approximately 12 nm (Figure 6(Bc)), indicating enhanced stability of the nanoparticles post-encapsulation. In addition, as shown in Supplementary Figure S3, the ζ potential of natural ferritin (NPLF) was −17.13 mV, and the absolute value of the potential of desferritin (Apo) was significantly reduced to −11.51 mV (32.7% decrease) due to the removal of the iron nucleus, and its colloidal stability was decreased (the DLS showed an increase in particle size, and slight aggregation was visible by TEM), which was consistent with the van der Waals force at |ζ| < 20 mV dominated aggregation; while, after encapsulating chlorogenic acid (CA), the ζ potential of Apo-CA was restored to −17.0 mV, which was close to the NPLF level, indicating that CA restored electrostatic repulsion through negatively charged group compensation and protein conformation rearrangement, which was consistent with the results of homogeneous particle size and no aggregation in the DLS/TEM, confirming that CA loading could effectively reverse the negative effect of deironing on the stability, and provide an opportunity for the functionalized design of ferritin carriers. It provides theoretical support for the functionalized design of ferritin carriers.

### 2.10. NPLF and Apo-CA Thermogravimetric Analysis

In this study, the upper-temperature limit for thermogravimetric analysis (TG) was set at 250 °C based on a combination of industrial practice and scientific validation: firstly, to cover extreme processing scenarios such as UHT sterilization (135–150 °C) and spray drying (200–250 °C), while preventing protein carbonation and harmful Maillard by-products (e.g., acrylamide). This threshold ensures the analysis of functional proteins relevant to food applications (denaturation, aggregation) with a 20% safety margin to buffer equipment fluctuations, followed by controlling the exposure time (5–20 °C/min ramp rate) and suppressing the risk of carbonation (below 280 °C onset temperature).

**Figure 7:**
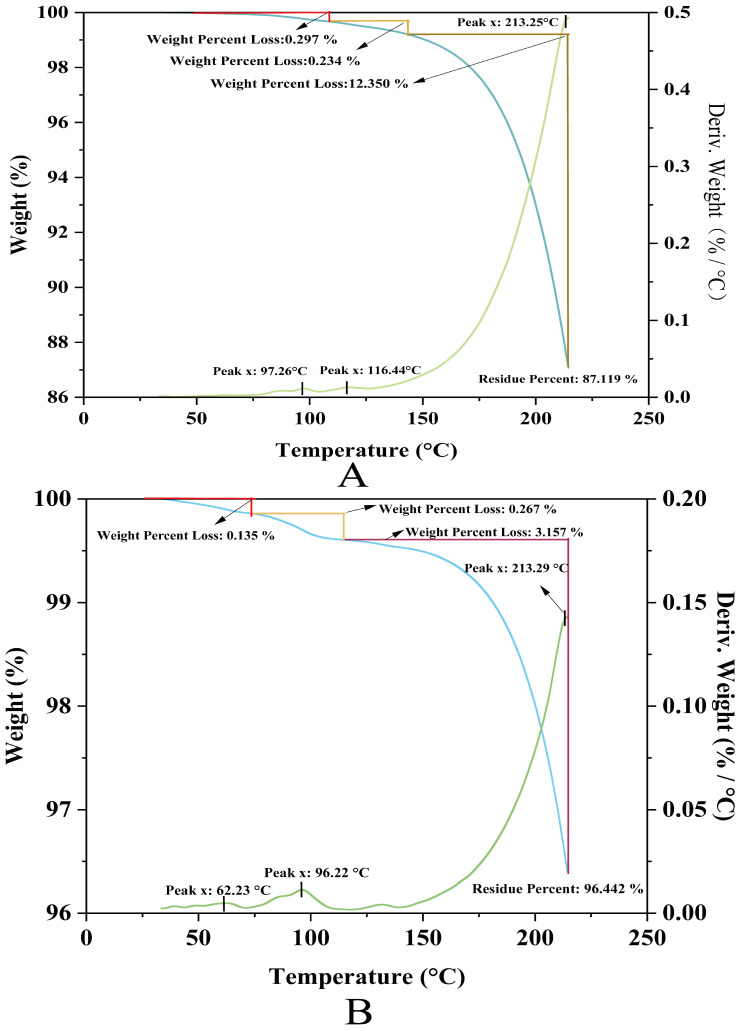
Thermogravimetry analysis of liver ferritin in northern pike. (**A**) NPLF; (**B**) Apo-CA.

Thermogravimetry analysis of ferritin revealed a three-stage thermal decomposition profile (Figure 7A). The minor mass loss (0.53% cumulative) at lower temperatures (97.26 °C and 116.44 °C) corresponds to the desorption of physiosorbed and weakly bound water molecules, indicating effective sample pretreatment with minimal residual moisture. The absence of significant mass variation below 200 °C confirms structural integrity within this range, supporting ferritin’s applicability in low-temperature processing systems. A pronounced mass reduction (12.35%) observed at 213.25 °C originates from thermal degradation of the protein shell, accompanied by volatile emissions (e.g., CO_2_, H_2_O, nitrogenous compounds). The high residual mass (87.1%) at 250 °C demonstrates exceptional thermal stability of the inorganic ferric core (likely Fe_3_O_4_ or FeOOH), highlighting ferritin’s hybrid organic–inorganic architecture: the protein shell confers biocompatibility while the mineral core ensures high-temperature inertness.

Comparative analysis demonstrated the superior thermal stability of Apo-CA (Figure 7B); the TG curves of Apo-CA showed that its thermal decomposition process was divided into three stages. The initial stage showed small weight loss (0.135%, 0.267%) around 62.23 °C and 96.22 °C, which might originate from the volatilization of adsorbed water or low-boiling solvents. The main decomposition peak was located at 213.29 °C, corresponding to a significant weight loss of 3.157%, suggesting thermal decomposition or functional group fracture of the material. The final residual rate was as high as 96.442%, demonstrating the excellent thermal stability of Apo-CA up to 250 °C and the ability to maintain the main structure intact at high temperatures.

### 2.11. Differential Scanning Calorimeter Analysis

**Figure 8:**
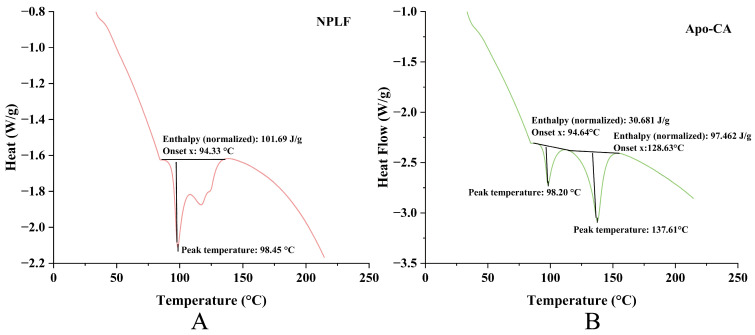
DSC analysis of liver ferritin in northern pike. (**A**) NPLF; (**B**) Apo-CA.

DSC analysis revealed remarkable thermal stability differences in ferritin before and after chlorogenic acid (CA) encapsulation. Pre-encapsulation, ferritin (Figure 8A) exhibited a single endothermic peak (peak temperature: 98.45 °C; enthalpy: 101.69 J/g), reflecting high structural thermal stability and a demand for substantial energy during the thermal transition. Post-encapsulation, Apo (Figure 8B) retained a thermal transition peak at a comparable temperature (98.20 °C) but with a notable enthalpy decrease (30.681 J/g), indicating that CA encapsulation disrupted the structural order of Apo, reducing its thermal stability. Moreover, the new endothermic peak at 137.61 °C in Figure 8B was hypothesized to relate to CA’s thermal behavior or novel structures formed via Apo-CA interactions, further highlighting encapsulation-induced thermal property alterations. This DSC evidence reinforces the TGA conclusion: the divergent thermal weight-loss behaviors of Apo-CA and NPLF in TGA—associated with iron content—were inherently supported by DSC-identified structural changes in Apo due to iron removal and CA encapsulation. Functioning complementarily, TGA illustrates macroscopic thermal weight-loss disparities, while DSC uncovers microscopic thermal transitions (e.g., protein denaturation enthalpy changes and CA-related new peaks). Together, they validate the influences of iron content, protein state, and encapsulation on material thermal behavior from the interconnected “composition-structure-thermal property” perspective [38].

## 3. Materials and Methods

### 3.1. Materials

The *Esox lucius*, which weighed 950 ± 50 g and was 45 ± 5 cm in length, was acquired from an aquatic market located in Uruqi, China. Dialysis bags (25 mm in width, Beijing Solabao Science and Technology Co.) (Beijing, China). Chlorogenic acid with 98% purity was acquired from Shanghai Yuanye Biotechnology Co. (Shanghai, China). Methanol (chromatographic grade) was purchased from Opson, Sweden; other analytically pure reagents, such as sodium chloride, were purchased from Tianjin Xinbute Chemical Co. (Tianjin, China).

### 3.2. Ultrasound-Assisted Extraction of Northern Pike Liver Ferritin

The ferritin extraction was carried out by using the technique that was reported by Zhang et al. [39], with some minor adjustments and modifications. The next step was to gather the fish livers, compress them until they were homogeneous, freeze-dry them, and store them at −20 °C. After that, 80 mL of K_2_HPO_4_ buffer (50 mM, pH 8.0) was mixed thoroughly with the homogenized and lyophilized liver samples. The resulting mixture was then subjected to ultrasonic processing using a 6 mm probe in an ultrasonic crusher (Scientz-950e, ScientzBiotechnology Co., Ltd., Ningbo, China) at an intensity of 20 kHz. The frequency was 20 kHz, the probe was a 6 mm flat head (pulse duration was 2 s on and 2 s off), and the probe was immersed in the liquid for 2 cm. The mixture was extracted and agitated at a temperature of 4 °C for a duration of 1 h. Subsequently, it was centrifuged twice at a speed of 10,000× *g* at 4 °C for 20 min each time. Immediately after removing the fat and sediment, the solution in the middle layer was collected. The liquid was heated to 60 °C for 20 min to eliminate heteroproteins while subsequently centrifuged at 10,000× *g* (4 °C) for 20 min. After that, a 50% saturated solution of (NH_4_)_2_SO_4_ was added to the supernatant. The protein was then left to settle by being kept in a chromatography cabinet at 4 °C for 10 h. Following the formation of the precipitate, the mixture underwent centrifugation at 10,000× *g* for 20 min at 4 °C. The leftover liquid had been subsequently discarded, and the red precipitate was gathered. Following the further dissolution of the precipitate in buffer (50 mM Tris-HCl, pH 7.5), a dialysis bag with a molecular weight of no more than 10 kDa was used to dialyze the mixture into purified water for 18 h. In the last step, the dialysate was subjected to centrifugation at a speed of 10,000× *g* for 15 min at a temperature of 4 °C. The resulting supernatant was then preserved. The chromatographic column underwent equilibration with five column volumes of 50 mM Tris-HCl (pH 9.0) before it was rinsed with a 50 mM Tris-HCl (pH 9.0) solution that included 0.15 M NaCl during an average flow rate of 1.5 mL/min. Finally, the absorbance of the eluent at 280 nm was determined.

### 3.3. Preparation of Apoferritin (Apo)

Ferritins that are capable of either removing iron nuclei artificially or not binding iron are referred to as Apoferritin (Apo). The method previously described by Pozzi was used to produce Apo [40]. The iron removal process focuses on removing iron from purified ferritin through three dialysis steps. Using 4 L of 20 mM Tris-HCI (pH 7.5) (containing 200 mM NaCl, 3 mM EDTA, and ammonium thioacetate (1:500) buffer, iron was first reduced and constricted. Afterward, any leftover ammonium thioacetate was eliminated using 4 L of 20 mM Tris-HCI (pH 7.5) (containing 200 mM NaCl). 20 mM Tris-HCl (pH 7.5) was eventually used to eliminate the remaining NaCl. Anaerobic conditions were used for all of the aforementioned procedures. ICP-MS (Agilent 7800, Agilent Technologies Co., Ltd., Colorado Springs, CO, USA) and Lowry’s technique were used to assess the iron content and protein concentration, respectively. 

### 3.4. Determination of Ferritin Content

The iron concentration within the final product has been examined using the phenanthrozine method [39], and a few changes have been implemented. The technique included the following steps: The sample solution (750 μL) was precisely measured and mixed with 250 μL associated with 10% trichloroacetic acid. Centrifugation was carried out during 4000× *g* for 2 min at a temperature of 4 °C. Subsequently, 650 μL of supernatant was collected post-centrifugation and combined with 100 μL of saturated ammonium acetate, 62.5 μL of 0.12 M ascorbic acid, and 62.5 μL of 0.25 M phenanthrozine. The final volume was adjusted to 1 mL using distilled water. Following a 2 h reaction period, the absorbance was quantified at 562 nm using a Cary50 spectrophotometer (Shanghai Spectrum Instrument Co., Ltd., Shanghai, China). The working curves of different concentrations of FeSO_4_-7H_2_O standard solution were plotted.

### 3.5. Single-Factor Experiments

Northern pike liver ferritin was extracted by ultrasonication using the method described in 3.2. The maximum values of ultrasonic power, ultrasonic time, and material–liquid ratio on ferritin extraction were evaluated separately (Table 6). The ultrasonic power was investigated at an extraction solution pH of 8.0, an extraction time of 25 min, and a material–liquid ratio of 1:4. The extraction time was evaluated at a fixed condition of ultrasound power of 200 W, pH of the extraction solution of 8.0 and material to liquid ratio of 1:4. The impact that this had on the material–liquid ratio on the extraction yield was examined at an ultrasonic power of 200 W, extraction time of 25 min, and pH of the extraction solution of 8.0.

### 3.6. Experimental Design of the Response Method

A Box–Behnken experimental design including three variables, ultrasonic power (X_1_), ultrasonic time (X_2_), and material–liquid ratio (X_3_), was used to determine the optimal combination of variables. The factors influencing the three levels of variability are shown in Table 7. Following the steps outlined in Section 3.2, the extraction procedure was executed, with the remaining conditions being an extract pH of 8.0 and an extract type of K_2_HPO_4_.

### 3.7. Protein Gel Electrophoresis

Both the sodium dodecyl sulfate-polyacrylamide gel electrophoresis (SDS-PAGE) and the natural polyacrylamide gel electrophoresis (Native-PAGE) of proteins were performed by using the technique that was reported by Zhang et al. [41], with some minor adjustments. An SDS-PAGE analysis was performed with a polyacrylamide separation gel that had 12% polyacrylamide and 5% polyacrylamide stacking at a steady current of 30 mA. For electrophoresis, samples weighing 10 micrograms were put on gel plates manufactured by Bio-Rad Laboratories in Hercules, California, US. The electrophoresis parameters were set as follows: Utilize 5 mA to operate the stacked gel for 50 min, followed by 15 mA till the front of the dye reaches the gel’s bottom.

A 5–20% gel has been used for Native-PAGE, with a current of 5 mA applied. Following electrophoresis, it was dyed by Coomassie Brilliant Blue R-250 for forty minutes, and then it washed out with twenty percent methanol and seven and a half percent acetic acid for an entire night. After that, the samples’ purity was assessed, and gel electrophoresis was used to measure the target protein’s molecular weight. Thyroglobulin 669 kDa, ferritin 440 kDa, bovine liver peroxidase protein 232 kDa, bovine heart lactate dehydrogenase protein 140 kDa, and bovine serum albumin 66 kDa were the five proteins that were mixed and labeled using Native-PAGE.

### 3.8. LC/MS Analysis

Lysate was added to the samples to give a final concentration of 1% SDC/100 mM Tris-HCl (pH = 8.5), and the protein concentration was determined by the BCA method after thorough mixing. An aliquot of protein was taken, and 1% SDC/100 mM Tris-HCl (pH = 8.5) solution was used to make up all samples to the same volume. After the addition of TCEP and CAA, incubate at 60 °C for 30 min to complete reduction and alkylation. An equal volume of ddH_2_O was added to dilute the concentration of SDC to less than 0.5%, trypsin was added at a mass ratio of enzyme to protein of 1:50, and the enzyme was incubated and oscillated overnight at 37 °C for digestion. The next day, TFA was added to terminate the digestion reaction, and the samples were centrifuged at 12,000× *g*. The supernatants were removed and desalted with a homemade SDB desalting column, vacuum-dried, and frozen at −20 °C. Samples were detected by mass spectrometry using an UltiMate 3000 RSLCnano nanolitre liquid tandem Q Exactive HF mass spectrometer from Thermo [42].

### 3.9. Homology Modeling

Swiss-Model Server (https://swissmodel.expasy.org/interactive/) (accessed on 15 March 2025) was used to finish the homology modeling process. The NPLF sequence was obtained by LC-MS/MS analysis. A template 3DI structure was obtained from PDB entry 3ajp, which may be accessed at https://www.rcsb.org (accessed on 15 March 2025). After constructing the NPLF model, the model was evaluated using Ramachandran plots and Verify-3D.

### 3.10. Preparation of Apolipoprotein-CA Nanoparticles Using the PH-Reversible Recombinant Properties of Ferritin

The reversible self-assembly property of ferritin was utilized to prepare Apoferritin-encapsulated chlorogenic acid nanoparticles (Apo-CA). Briefly, the pH of 1 μM Apo was first adjusted to 2.0 with 1 M HCl, at which time ferritin was broken down into individual subunits in the acidic environment of pH 2.0, and then a specific concentration of CA solution was gradually introduced to the Apo solution. The resultant solution underwent stirring at a temperature of 4 °C for 30 min before the gradual adjustment of the pH to 7 using 0.5 M NaOH. The mixture of solutions had been stirred at 4 °C for 20 min before being stored in a dark atmosphere for 3 h. To remove free CA from the NPLF solution, the solution was dialyzed four times in a 20 mM Tris-HCl (pH 7.5) buffer. After that, the produced Apo-CA solution was kept at 4 °C in the dark.

### 3.11. High-Performance Liquid Chromatography Analysis

The CA content was analyzed by a high-performance liquid chromatography (HPLC) system (Shimadzu, Kyoto, Japan) with a UV-visible detector and a C18 column (5 μm, 250 × 4.6 mm; Shimadzu, Japan). The production of a standard curve for CA involves the use of a variety of CA solutions with varied concentrations. The sample was extracted using a 0.4% phosphoric acid/acetonitrile (87:13, *v*/*v*) mobile phase, 40 μL injection volume, 1.0 mL/min flow rate, and 327 nm wavelength. The pH of the samples was lowered to below 2.0 to disrupt the structure of Apo-CA and release CA. The determination of the encapsulation efficiency of CA in Apo-CA was conducted utilizing Equation (2).

(2) Encapsulation rate=Released CA/Encapsulated CA×100%

### 3.12. Fourier Transform Infrared (FTIR) Spectral Analysis

Fourier transform infrared (FTIR) spectra of NPLF, Apo, and Apo-CA were analyzed using NicoletiS10 (Thermo FisheScientific, Waltham, MA, USA). The lyophilized sample powder was combined with potassium bromide powder in a ratio of 1:100 and milled into a uniform powder, covering a spectral range from 4000 cm^−1^ to 400 cm^−1^.

### 3.13. Ultraviolet-Visible Absorption Spectroscopy Analysis

The UV-visible spectra of NPLF, Apo, and Apo-CA solutions at 200–400 nm wavelengths were determined by a UV spectrophotometer and analyzed for comparison [43]. Every sample was subjected to the same treatment, and the blank control solution consisted of Tris-HCl buffer.

### 3.14. Intrinsic Fluorescence Spectral Analysis of Samples

The fluorescence spectra of NPLF, Apo, and Apo-CA were collected using a 970CRT fluorescence spectrophotometer (INESA Analytical Instruments Co., Ltd., Shanghai, China) [44]. The excitation and emission slit widths were configured to 5 nm each. The excitation wavelength utilized was 290 nm, and data were collected in the range of 300 to 500 nm.

### 3.15. Transmission Electron Microscopy Experiments

Firstly, each sample was placed on a clean sealing film, and then the copper mesh covered by the carbon film was placed face down on the sample for 5 min; then, the extra liquid was removed from the edge using filter paper. Next, we negatively stained the mass fraction for 3 min using a 2% phosphotungstic acid solution, sucked off the excess stain, dried the samples, and then observed and imaged them using a transmission electron microscope at 80 kV (HT7700, Hitachi, Tokyo, Japan). The samples were observed and imaged by an 80 kV (HT7700, Hitachi, Tokyo, Japan) transmission electron microscope.

### 3.16. Dynamic Light Scattering Experiment and ζ-Potential Analysis

Hydrodynamic radius variations and ζ potentials of NPLF, Apo, and Apo-CA were evaluated according to a modified version of the method outlined by Yang et al. [45]. Hydrodynamic radii and ζ-potentials of NPLF, Apo, and Apo-CA samples were measured using a Malvern Nano ZS90 (Malvern Instruments Ltd. Malvern, UK) measured at an angle of 90°. In the case of dynamic light scattering, undiluted samples (1 mL) were analyzed in disposable cuvettes with a path length of 10 mm. The equilibration time was set to 120 s. Each sample was recorded 3 times for a total of 14 runs. The analytical model was Malvern Software (Zetasizer Software 8.02) General Purpose (normal resolution). ζ-potential analysis was performed with a DTS1070 cell. ζ-potential analytical model was set to Malvern Software’s automatic mode. All measurements were performed at 25 °C and each value was taken in triplicate. All sample solutions were filtered through a 0.22 μm hydrophilic membrane to minimize impurity interferences, and three sets of data were measured for each sample, with the average of three replicate measurements calculated and plotted using OriginPro 2022 software.

### 3.17. Thermogravimetry Analysis

The TG technique allows for the simultaneous determination of the thermal stability and thermal effects of materials, enabling efficient and simultaneous analysis of the thermal behavior of the materials and improving the accuracy and efficiency of the experiments. The NPLF and Apo-CA samples were accurately weighed and placed in alumina crucibles, protected by a nitrogen atmosphere (flow rate of 50 mL/min), and ramped up at a constant rate of 5 °C/min from room temperature to 200 °C. Before testing, the instrument was calibrated using standard substances (sapphire, indium, zinc) for temperature and heat flux. The instrument was calibrated for temperature and heat flow using standards (sapphire, indium, zinc), and the mass change and heat flow signals were captured in real time by STARe software V17.x. After the baseline correction, the experimental data were integrated and analyzed using OriginPro 2022 for thermogravimetric (TG).

### 3.18. Differential Scanning Calorimeter Analysis Experiment (DSC)

The NPLF and Apo-CA samples were accurately weighed and placed in an alumina crucible, and then the instrument was calibrated for temperature and heat flow by using indium, zinc, and other standard substances; during the test, the temperature was increased from room temperature to 200 °C under a protective atmosphere of 50 mL/min nitrogen gas at a constant rate of 5 °C/min, and the heat flow signals were captured in real-time by using the STARe software; the baseline calibration was performed on the raw data after the experiment, and the differential heat flow curves (DSC curves) were plotted by using OriginPro 2022. At the end of the experiment, the raw data were baseline corrected, the differential heat flow curve (DSC curve) was plotted by using OriginPro 2022, and the enthalpy change values were calculated by identifying the onset and peak temperatures of the heat-absorbing/exothermic peaks and integrating them, and the phase transition temperature and energy change process of the materials were finally characterized [46].

### 3.19. Data Analysis

All of the experiments that were carried out for this research were carried out three times, and the results were provided as the mean and the standard deviation (SD). Statistical analyses utilized SPSS 17.0 software, with a significance threshold established at *p* < 0.05. NPLF structures were analyzed through visualization in PyMol.

## 4. Conclusions

In this study, homogeneous monodisperse ferritin (NPLF) was successfully produced from northern pike liver in a yield of 139.46 mg/kg by optimizing the ultrasonic-assisted extraction process (200 W power, 1:3 material–liquid ratio, and 25 min treatment). Structural characterization showed that NPLF was a caged nanoparticle (molecular weight of 480 kDa, substituent of 19 kDa) that formed a three-dimensional structure through a four-helix bundle (A-D) and a three-dimensional structure was formed by a four-helix bundle (A-D) and short helix E. DLS and TEM confirmed that the hydrated particle size was stable at 12 nm. ζ potential analysis showed that the surface potential of natural NPLF was −17.13 mV, and the absolute value of Apo after iron removal (Apo) plummeted by 32.7% to −11.51 mV, which resulted in the deterioration of colloidal stability (DLS showed an increase in particle size, and aggregation was visible in TEM), which was consistent with the fact that van der Waals forces dominated the aggregation at |ζ| < 20 mV. mV, van der Waals forces dominate aggregation; whereas, after chlorogenic acid (CA) encapsulation, the Apo-CA potential is restored to −17.0 mV, and the electrostatic barrier is re-established through the compensation of negatively charged groups and conformational rearrangement, which is corroborated by the homogeneous particle sizes and the absence of aggregation in DLS/TEM. The CA encapsulation achieved based on pH-reversible self-assembly properties (13.25% loading rate, determined by HPLC on a C18 reversed-phase column) was characterized by a triple stabilization mechanism: (1) the 8 nm hydrophobic cavity isolates ROS/hydrolysis enzymes through a physically confined domain, and the DLS particle size remained at 12 nm after loading; (2) the CA benzene ring binds to the hydrophobic residues, and phenolic hydroxyl group forms a hydrogen-bonding network with the polar residues (Glu/Asn), which is a key factor in the stabilization of DLS. Fluorescence burst analysis showed static burst dominance and enhanced rigidity of non-covalent interactions; (3) TG/DSC confirmed that the thermal stability of the drug-loaded complex was significantly enhanced. This multidimensional stabilization strategy of physical restriction-hydrophobic hydrogen bonding synergy-thermodynamic optimization not only reverses the stability damage caused by iron removal but also confers excellent biostability to Apo-CA, which highlights the potential of barracuda ferritin in the field of nutrient delivery and provides a theoretical paradigm for the development of marine ferritin resources.

## Figures and Tables

**Table 1:** Box–Behnken design matrix.

NO.	X_1_/Ultrasonic Power (W)	X_2_/Ultrasound Time (min)	X_3_/Solid–Liquid Ratio	Y/The Amount of Ferritin Extracted (mg/kg)	
				Measured Value	Predicted Value
1	200	25	1:3	136.21 ± 2.72	135.65
2	300	25	1:2	84.22 ± 1.68	81.94
3	100	25	1:4	87.8 ± 1.76	90.08
4	200	20	1:2	91.06 ± 1.82	89.30
5	200	30	1:2	101.48 ± 2.03	103.81
6	100	25	1:2	95.66 ± 1.91	97.38
7	200	25	1:3	135.29 ± 2.71	135.65
8	200	30	1:4	92.59 ± 1.85	94.36
9	200	25	1:3	134.85 ± 2.70	135.65
10	200	20	1:4	116.74 ± 2.33	114.41
11	100	20	1:3	120.21 ± 2.40	120.26
12	200	25	1:3	132.45 ± 2.65	135.65
13	200	25	1:3	139.46 ± 2.79	135.65
14	300	30	1:3	117.22 ± 2.34	117.17
15	300	20	1:3	92.59 ± 1.85	94.36
16	300	25	1:4	106.61 ± 2.13	104.89
17	100	30	1:3	98.23 ± 1.96	94.18

Note: Values are given as mean ± standard deviation.

**Table 2:** Analysis of variance for the second-order polynomial model.

Source	Sum of Squares	df	Mean Square	F Value	*p* Value
Model	5966.95	9	662.99	50.44	<0.0001
X_1_/Ultrasonic power	0.20	1	0.20	0.015	0.9057
X_2_/Ultrasound time	15.35	1	15.35	1.17	0.3157
X_3_/Solid–liquid ratio	122.62	1	122.62	9.33	0.0185
X_1_X_2_	543.12	1	543.12	41.32	0.0004
X_1_X_3_	228.77	1	228.77	17.40	0.0042
X_2_X_3_	298.77	1	298.77	22.73	0.0020
$x_{1}^{2}$	1325.42	1	1325.42	100.83	<0.0001
$x_{2}^{2}$	495.42	1	495.42	37.69	0.0005
$x_{3}^{2}$	2493.90	1	2493.90	189.72	<0.0001
Residual	92.02	7	13.15		
Lack of Fit	66.18	3	22.06	3.41	0.1332
Pure Error	25.84	4	6.46		
Cor Total	6058.96	16		50.44	50.44
R^2^ = 0.9848			$R_{adj}^{2}$ = 0.9653		

**Table 3:** Main peptide sequences of NPLF.

No.	Amino Acid Sequence	Amino Acid Number	Theo. MH + [Da]
1	IFLQDIK	7	875.51165
2	TVNQALLDLHK	11	1250.6983
3	IAADKVDPHLCDFLETHYLGEQVEAIK	27	3110.5383
4	VDPHLCDFLETHYLGEQVEAIK	22	2612.2581
5	MAEYLFDK	8	1015.4685
6	DDVALPGFAHFFK	13	1462.7245
7	FLAFQNK	7	866.46504
8	DEWGSGLEAMQCALQLEK	18	2063.9292
9	IAADKVDPHLCDFLETHYLGEQVEAIKK	28	3238.6332
10	KPDRDEWGSGLEAMQCALQLEK	22	2560.205

**Table 4:** Iron removal rate of ferritin in Northern Pike ICP-MS.

Working Parameters	Setpoint
Pump Rate	20 r/min
Nebulizer Flow	1.00 L/min
Auxiliary Gas	1.00 L/min
Sample Flush Time	40 s
RF Power	1550 w

**Table 5:** Ferritin excretion rate in northern pike.

Working Parameters	Iron Content Before Deferring (μg/L)	Iron Content After Decentralization (μg/L)	Iron Removal Rate (%)
northern pike	7259.22 ± 18.51	393.17 ± 1.8	94.58

Note: Values are given as mean ± standard deviation.

**Table 6:** Factors influencing the amount of ferritin extracted.

Factor	Level				
Extract pH	7.0	7.5	8.0	8.5	9.0
Ultrasonic power (W)	100	200	300	400	500
Ultrasound time (min)	10	15	20	25	30
Solid–liquid ratio	1:2	1:3	1:4	1:5	1:6

**Table 7:** Factors and their levels employed in Box–Behnken design.

Factor	Level		
	−1	0	1
X_1_/Ultrasonic power (W)	100	20	1:2
X_2_/Ultrasound time (min)	200	25	1:3
X_3_/Solid–liquid ratio	300	30	1:4

## Data Availability

Data are contained within the article and Supplementary Materials.

## Supplementary Materials

The following supporting information can be downloaded at: https://www.mdpi.com/article/10.3390/molecules30092080/s1, Figures S1–S3: Effect of different buffer types on the amount of ferritin extracted (without the addition of the sonication method); The verify-3D plot of NPLF; Zeta potential analysis of different samples (NPLF, Apo, Apo-CA); Tables S1–S6: Raw data on ultrasound power; ANOVA experimental analysis of ultrasound power; Duncan and Waller Duncan tests for ultrasonic power; Raw data for solid-liquid ratio; ANOVA experimental analysis of solid-liquid ratio; Duncan and Waller Duncan tests for solid-liquid ratio.
