# Supplementary material for: Ultrasound-Assisted Ferritin Extraction from Northern Pike Liver: An Innovative Approach for Chlorogenic Acid Encapsulation with Enhanced Thermal Stability
Source: Molecules. 2025 May 7;30(9):2080. doi: 10.3390/molecules30092080 (PMC12073890; doi:10.3390/molecules30092080)
Supplement: Supplementary file 1 [file molecules-30-02080-s001.zip › molecules-3596882-supplementary.pdf]

	Ultrasonic power				
	100W	200W	300W	400W	500W
1	128.36	135.19	116.18	62.25	41.08
2	126.41	129.53	117.44	61.47	41.29
3	127.37	132.51	116.81	61.86	41.98

Table S1 Raw data on ultrasound power

ANOVA

Ferritin extraction

			square sum	degrees of freedom	mean square	F	significance
interclass	(Combined)		20654.06724	4	5163.517	1432.098	0.000
	linear term	compare	17628.78243	1	17628.782	4889.333	0.000
		deviation	3025.28481	3	1008.428	279.687	0.000
	Quadratic term	compare	1744.59705	1	1744.597	483.863	0.000
		deviation	1280.68776	2	640.344	177.599	0.000
	cubic term	compare	913.119	1	913.119	253.253	0.000
		deviation	367.569	1	367.569	101.945	0.000
	Fourth-order term	compare	367.569	1	367.569	101.945	0.000
intra-class			36.056	10	3.606		
Total			20690.12284	14			

Table S2 ANOVA experimental analysis of ultrasound power

Ferritin extraction							
Ultrasound power		Number of cases	Subset of Alpha = 0.05				
			1	2	3	4	5
Duncana	500	3	41.45				
	400	3		61.86			
	300	3			116.81		
	100	3				127.38	
	200	3					132.41
	significance		1	1	1	1	1
Waller Duncana,b	500	3	41.45				
	400	3		61.86			
	300	3			116.81		
	100	3				127.38	
	200	3					132.41
The mean of each group in the chi-subset will be shown.							
a Using the reconciled mean sample size = 3.000.							
b Type I/II error severity ratio = 100.							

Table S3 Duncan and Waller Duncan tests for ultrasonic power

	Solid-liquid ratio				
	1：2	1：3	1：4	1：5	1：6
1	103.03	137.95	137.95	136.73	109.78
2	104.73	141.29	141.29	133.54	112.20
3	105.89	139.14	139.14	137.22	111.26

Table S4 Raw data for solid-liquid ratio

ANOVA

Ferritin extraction

			square sum	degrees of freedom	mean square	F	significance
interclass	(Combined)		3155.91	4	788.977	384.513	0.000
	linear term	compare	26.696	1	26.696	13.011	0.005
		deviation	3129.213	3	1043.071	508.347	0.000
	Quadratic term	compare	2853.347	1	2853.347	1390.597	0.000
		deviation	275.866	2	137.933	67.222	0.000
	cubic term	compare	56.994	1	56.994	27.776	0.000
		deviation	218.872	1	218.872	106.669	0.000
	Fourth-order term	compare	218.872	1	2218.872	106.669	0.000
intra-class			20.519	10	2.052		
Total			3176.428	14			

Table S5 ANOVA experimental analysis of solid-liquid ratio

Ferritin extraction						
solid-liquid ratio		Number of cases	Subset of Alpha = 0.05			
			1	2	3	4
Duncan's test	2	3	104.55			
	6	3		111.08		
	4	3			135.7	
	5	3			135.8	
	3	3				139.46
	significance		1	1	0.898	1
Waller-Duncan test	2	3	104.55			
	6	3		111.08		
	4	3			135.7	
	5	3			135.8	
	3	3				139.46
The mean of each group in the chi-subset will be shown.						
a Using the reconciled mean sample size = 3.000.						
b Type I/II error severity ratio = 100.						

Table S6 Duncan and Waller Duncan tests for solid-liquid ratio

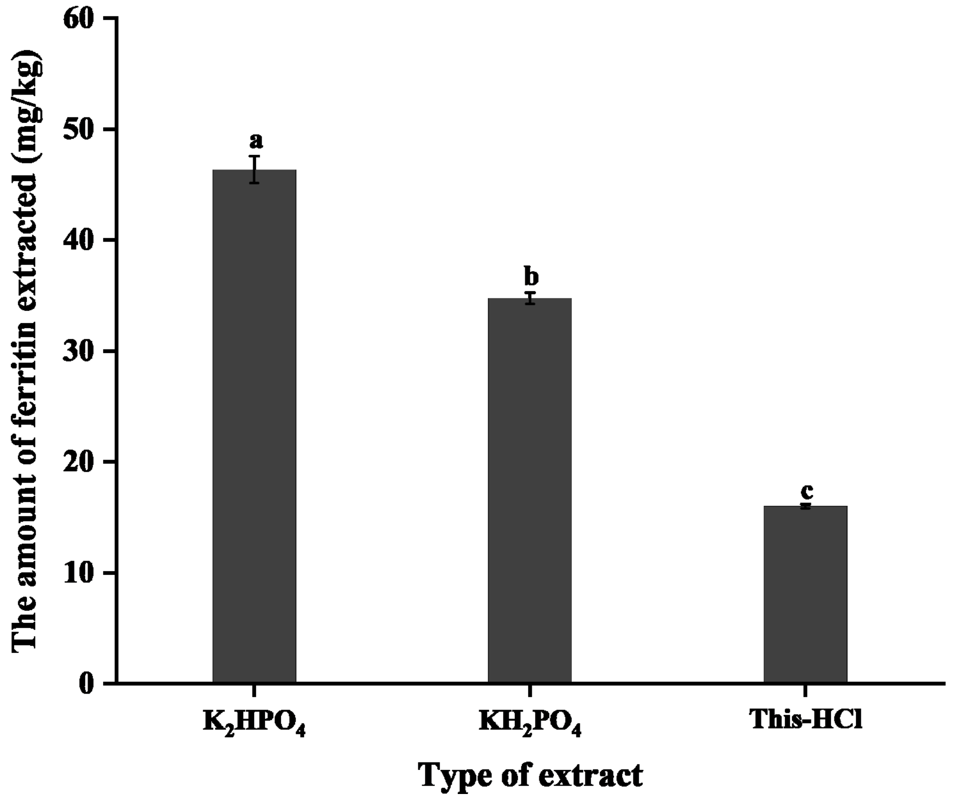

Figure S1 Effect of different buffer types on the amount of ferritin extracted (without the addition of the sonication method)

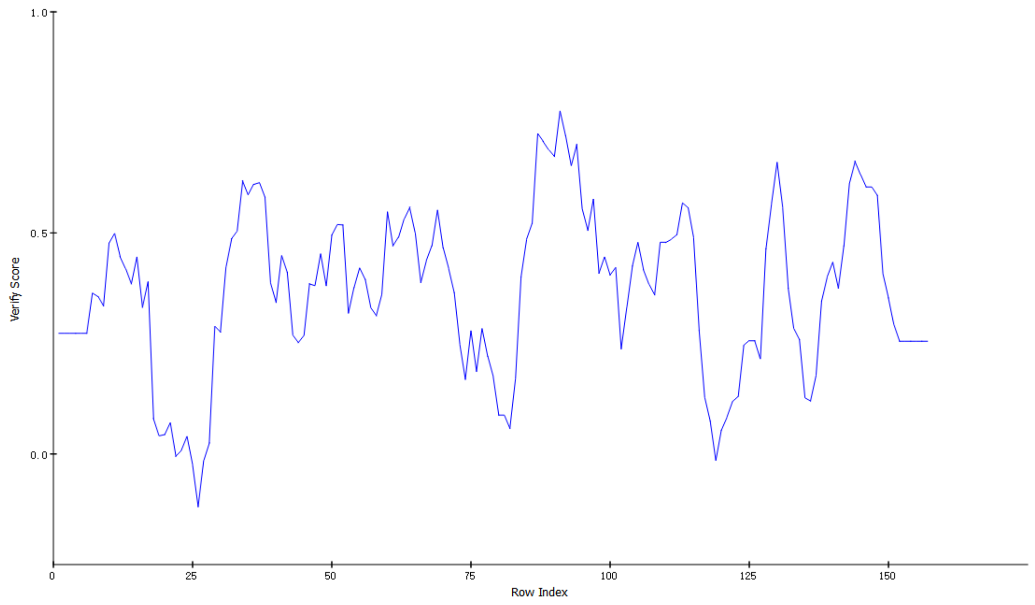

Figure S2 The verify-3D plot of NPLF

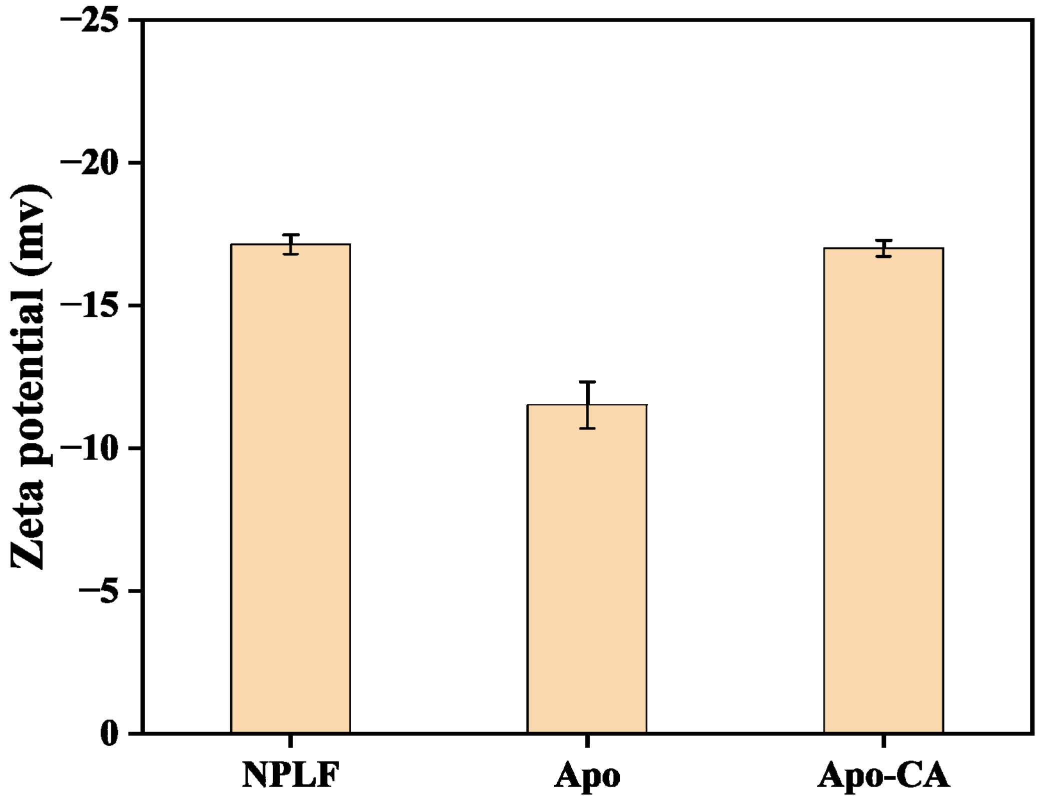

Figure S3 Zeta potential analysis of different samples (NPLF, Apo, Apo-CA)
